# The orthodontic-endodontic interface: trauma and pulpal considerations

**DOI:** 10.1038/s41415-024-7786-9

**Published:** 2024-09-13

**Authors:** Peter Parashos

**Affiliations:** Melbourne Dental School, Faculty of Medicine, Dentistry and Health Sciences, The University of Melbourne, Victoria, Australia

## Abstract

The interpretation of the clinical signs and symptoms arising from the interdisciplinary relationship between orthodontics and endodontics becomes more complicated when superimposed by dental trauma. A history of dental trauma before or during orthodontic tooth movement may have implications for pulpal health and clinical outcomes. An understanding of the biology is essential for appropriate treatment planning. This review and treatment recommendations will assist dental practitioners in managing orthodontic-endodontic interactions.

## Key points

- The biological interactions between the disciplines of orthodontics and endodontics are many and varied.
- The dental pulp will be affected by any orthodontic tooth movement, with the biological outcome dependent on the type and degree of orthodontic forces.
- Deciding on appropriate orthodontic rest periods should be based on both pulpal and periodontal responses to the type and severity of the trauma sustained.
- Clinicians should be able to base clinical decisions about the interactions between the two disciplines on the information provided in this review.

## Introduction

An appreciation of the biological basis and implications of the relationships between the disciplines of orthodontics and endodontics is important for dental practitioners. Previous reviews have covered a variety of different interactions,^1^^,^^2^ but the two more complex relationships are the orthodontic management of traumatised teeth and the pulpal response to orthodontic tooth movement (OTM). This narrative review will explore the biological and mechanical aspects of the relationship between OTM, dental trauma and the pulp; it is based on a recent review of a variety of topics, including pulp biology, tooth resorptions, root-filled teeth, traumatised teeth, endodontic treatment during orthodontics and OTM during endodontic treatment.^2^

## Orthodontics and dental trauma

Loss of pulp vitality, root resorption and pulp canal calcification (PCC) have been identified as the main risks of OTM for teeth with a history of trauma.^3^ These risks are reviewed below.

### Pulp necrosis

Early literature reported a link between OTM and pulp necrosis of teeth with a history of dental trauma,^4^^,^^5^^,^^6^ and two systematic reviews reached the same conclusion.^7^^,^^8^ However, other reviews reported that there was no such evidence.^9^^,^^10^^,^^11^ Of the main studies that reported an increased risk,^12^^,^^13^^,^^14^^,^^15^^,^^16^ Brin *et al*.^12^ based their conclusion only on the non-response to electric pulp tests and hence the diagnosis of loss of vitality is doubtful. On the other hand, in the research by Bauss *et al*.,^13^^,^^14^^,^^15^^,^^16^ the criteria included loss of pulpal sensibility, grey discolouration and periapical radiolucency. These authors concluded that traumatised maxillary incisors with severe periodontal injuries were more susceptible to pulp necrosis than non-traumatised teeth after intrusive orthodontic forces.^14^ A similar conclusion was reported for intrusive OTM in teeth traumatised during OTM,^15^ traumatised teeth undergoing extrusive OTM,^16^ and teeth with total PCC.^13^ The authors also hypothesised that a history of previous severe periodontal ligament (PDL) damage may somehow have resulted in teeth being less resistant to extrusive OTM due to impaired pulpal blood flow, and therefore light orthodontic forces were indicated.^16^

In summary, while some reviews report insufficient evidence of increased risk of pulp necrosis with OTM of traumatised teeth,^9^^,^^10^^,^^11^ those reviews were based on literature with significant methodological problems.^2^^,^^7^^,^^17^ Therefore, there is indeed some evidence to suggest that a history of previous trauma may predispose a tooth to pulpal necrosis after OTM and it may be influenced by the type and severity of orthodontic forces.^13^^,^^14^^,^^15^^,^^16^

### Root resorption

**Fig. 1:**
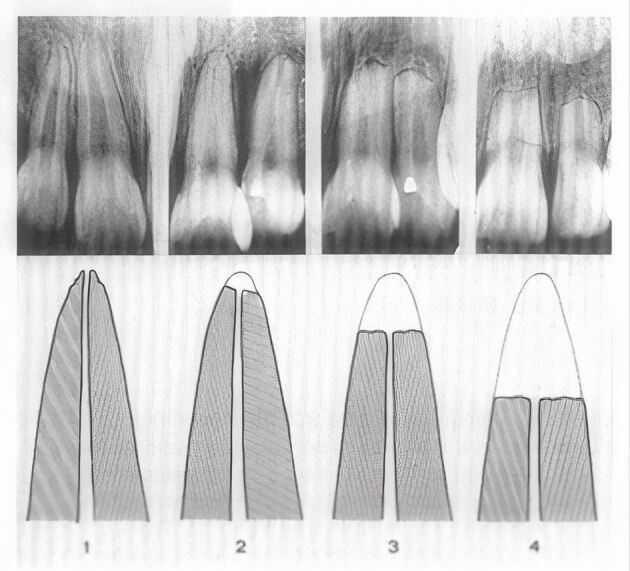
Index for quantitative assessment of apical root resorption: 1) irregular root contour; 2) resorption <2 mm of the original root length; 3) resorption 2 mm to 1/3 original root length; 4) resorption > 1/3 original root length. Reprinted from *American Journal of Orthodontics*, Vol 82, Malmgren *et* a*l*., ‘Root resorption after orthodontic treatment of traumatized teeth' pp 487-491, Copyright 1982, with permission from Elsevier^27^

Review articles have concluded that the studies reporting the effect of a history of dental trauma on root resorption related to OTM are very few in number and conflicting.^3^^,^^9^^,^^10^^,^^11^^,^^18^^,^^19^^,^^20^^,^^21^^,^^22^^,^^23^ Two early reports cited in these reviews provided no real evidence of a history of dental trauma,^24^^,^^25^ while another early article concluded that avulsed or ‘partially avulsed' teeth were more likely to show apical root resorption after OTM.^26^ In 1982, Malmgren *et al*.^27^ retrospectively assessed the prevalence and extent of root resorption in 55 incisor teeth in 27 patients aged 7-15 years with a previous history of ‘slight or moderate' trauma. From intraoral radiographs before and after OTM, signs of root resorption of the teeth were classified based on a resorption index (Fig. 1). The authors concluded that slight or moderate dental injuries did not have a greater risk of root resorption than uninjured teeth and there was no difference between the types of mild-moderate injury.^27^ Further, previously traumatised teeth with signs of apical root resorption before OTM may be more prone to root resorption during treatment.^26^^,^^27^

In a large study of 2,451 maxillary incisors in 719 young orthodontic patients, the average loss of root length for the traumatised teeth (type and severity not specified) was 1.07 ± 1.19 mm compared with 0.64 ± 1.04 mm for the non-traumatised teeth.^28^ While the accuracy of the measurements and the uncertainty of the trauma history were limitations, the amount of resorption was not clinically significant. In a subsequent study on 485 of those 719 patients,^29^ the authors confirmed that a history of trauma more than one year before the OTM was the most important predictor of apical root resorption, albeit that the precise trauma history was unknown.

Brin *et al*.^12^ reported that moderate root resorption (Grades 1-2 of the Malmgren *et al*. classification)^27^ was observed in 27.8% of previously traumatised teeth compared with 7.8% in the OTM-only group and 6.7% in the trauma-only group. A study in young patients reported no statistically significant correlation between a history of trauma and apical root resorption.^30^ However, pausing OTM for 2-3 months resulted in significantly less resorption (0.4 ± 0.7 mm) than continuous OTM (1.5 ± 0.8 mm).^30^ Another study assessed the effect of different orthodontic archwire sequences on apical root resorption and reported that a history of incisor tooth trauma did not increase the risk of resorption.^31^ However, the sample sizes in that study were small and the authors warned that the results were not compelling.^31^

Several other recent studies have also reported a lack of a convincing relationship between apical root resorption and a history of trauma subsequent to OTM, mainly due to unreliable data as reported by the authors.^32^^,^^33^^,^^34^^,^^35^^,^^36^ Importantly, the Malmgren index based on two-dimensional radiographs may not be accurate for cone beam computed tomography (CBCT) imaging.^33^ Also, case reports on the effect of OTM on traumatised immature teeth after apexification or treated with regenerative endodontic procedures, showed no increased predisposition to apical root resorption.^37^^,^^38^^,^^39^^,^^40^^,^^41^ Instead, the teeth were more at risk of pulp necrosis or different types of root resorption. However, the knowledge base relating to this novel trauma literature is very limited at this time.

Overall, the literature in this area suffers from small sample sizes, large standard deviations, lack of information on the intensity, location and type of trauma, potentially inaccurate measurement methods of the root lengths, and relatively short timeframes of the assessments. Recent reviews have identified a broad range of variables that may potentially affect the relationship between root resorption and OTM.^42^^,^^43^ Notwithstanding these issues, it appears that there is no convincing evidence to indicate that teeth with a history of trauma are routinely at greater risk of apical root resorption after OTM. The evidence supporting this contention demonstrated clinically non-significant degrees of resorption and it may relate to more severe forms of trauma, particularly affecting the PDL. However, pre-OTM evidence of apical root resorption may be predictive of an increased risk of apical root resorption during OTM.

### Pulp canal calcification

The term pulp canal calcification (PCC) reflects a more accurate radiographic representation of the biological process than the term pulp canal obliteration.^2^ Historically, ‘calcified obliteration of the pulp-chamber' was first reported in 1905.^44^ However, complete calcification of the pulp chamber subsequent to trauma and OTM was first reported in 1950.^45^ Another case report^46^ showed complete calcification of a replanted iatrogenically avulsed immature mandibular premolar. In 1966, Andreasen and Hjørting-Hansen^47^ published a landmark paper on the outcomes of 110 replanted teeth including 13 immature incisors, of which seven showed gradual ‘obliteration' of the pulp chamber. This gradual calcification was attributed to ‘pulpal damage' that Andreasen^48^ later described as an ‘accelerated speed of dentine deposition', resulting in either partial or total calcification.^49^ The calcification was suggested to be a sequel to revascularisation and/or reinnervation of the entire traumatised pulp or parts of it.^50^^,^^51^ A recent literature review discusses the possible mechanisms leading to PCC.^2^

Pulp necrosis subsequent to PCC is an uncommon complication, with a prevalence in the range of 1-27.2%^52^^,^^53^ over observation periods of 3.4-16 years,^53^ This prevalence seems to increase over time.^48^^,^^54^^,^^55^ Possibly because of the continuing PCC, subsequent injuries may sever the vascular supply at the narrowing apical foramen.^48^ New injuries to the pulp in a tooth with PCC may be in the form of restorative procedures or direct trauma.^54^^,^^55^ Another suggested cause was orthodontic treatment,^56^ possibly due to the greater risk of damage to the vascular supply during OTM,^13^^,^^15^ which may be considered to be a controlled^57^ or subtle^58^ form of trauma to teeth. The evidence linking PCC to OTM is discussed below.

#### Radiographic evidence

Delivanis and Sauer^59^ questioned whether PCC was an iatrogenic side effect of orthodontic treatment or simply an isolated problem. Of 46 patients studied, only two showed PCC after OTM and both were subjected to heavy OTM forces or OTM over a very long period of time. Despite this evidence, the authors cautiously concluded that the possibility of PCC should be closely monitored in patients undergoing OTM or those in retention. On the other hand, another study^60^ reported that PCC was radiographically more prevalent in orthodontic patients (17%) than in a control group (8%); although, there were no details provided concerning a trauma history or other treatment details. In another early radiographic study, Popp *et al*.^57^ observed evidence of PCC in incisor teeth of patients in both the experimental and control groups. They interpreted this as a normal ageing process, which may have been due either to rest periods of the OTM allowing tissue repair, or that the forces were within physiological limits. However, a study on palatally impacted canines concluded that excessive OTM resulted in PCC.^61^ Three retrospective studies reported significant increases of 2.2%,^62^ 4%^63^ and 22%^64^ in the prevalence of pulp stones in teeth after OTM; although, the studies lacked control groups and were based on panoramic radiographs. A novel CBCT study reported a significant decrease in pulp volume of maxillary anterior teeth after some 18 months of OTM.^65^ The initial pulp volumes ranged from 38.12-51.02 mm^3^ with mean volume losses after OTM varying from 3.04-3.86 mm^3^. More recent literature concluded that OTM significantly increased the prevalence of pulp stones and pulp calcification.^66^^,^^67^^,^^68^ In one study, there was a significant increase in the number of pulp stones of some 14% compared with 3% for the non-OTM group.^66^ The other studies reported a 38% increase in the number of pulp stones in the post-OTM treatment radiographs^67^ and a prevalence of pulp stones and PCC in molars of some 42%.^68^

#### Histological evidence

**Fig. 2:**
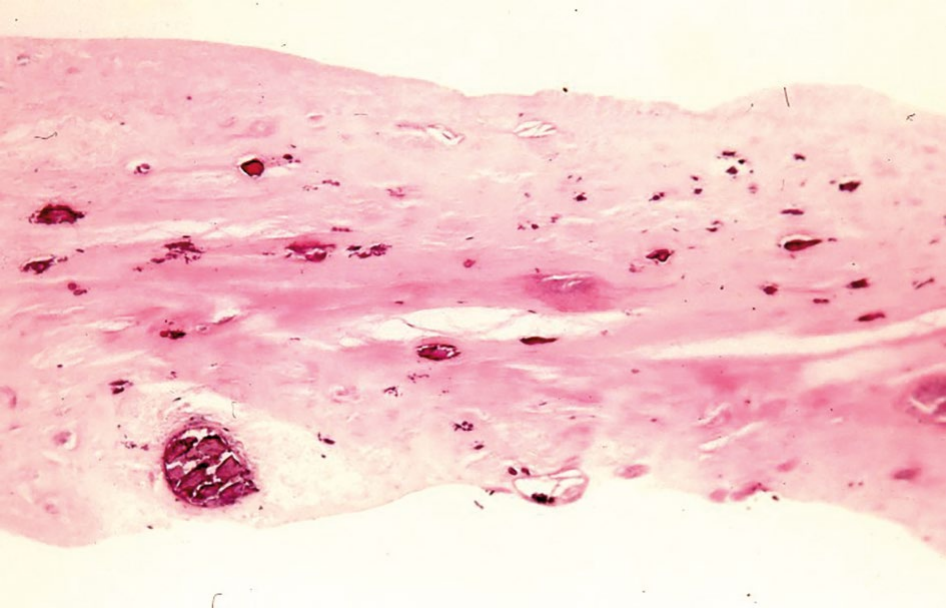
Histological section of pulp from a maxillary incisor tooth with a history of luxation followed several weeks later by avulsion and immediate replantation. Pathology report indicated ‘collections of microorganisms in coccal and filamentous forms. Dystrophic calcification of varying sizes throughout the necrotic tissue. Necrotic infected pulp' (H&E x 33). Reproduced from Parashos P, ‘Endodontic-orthodontic interactions: a review and treatment recommendations' *Australian Dental Journal*, Vol 68, 2023, Wiley^2^

In an early study, orthodontic intrusion was not linked to the frequency of pulp stones in children; although, the research protocol lacked standardisation of the forces and the number of days over which the forces were applied.^69^ Several other histological studies^70^^,^^71^^,^^72^ reported varying findings depending on the degree and duration of applied forces, which indicated an inconsistent relationship between OTM forces, timeframes and prevalence of pulp calcifications. When calcification was present, in the coronal pulp it appeared as discrete and concentric pulp stones, but in the radicular pulp was more diffuse.^71^ When the pulp calcifications were large and numerous, they could occupy part or all of the pulp cavity.^71^ Therefore, it is feasible that the continued growth of the calcifications could lead to their coalescence resulting in PCC radiographically. Mild to moderate trauma can result in dystrophic calcification which may result in PCC unless the pulp becomes completely necrotic (Fig. 2). However, the exact mechanism by which pulp calcifications coalesce is unclear and is complicated by the different types of pulp stones and calcifications identified histologically.^73^

In summary, recent review papers indicate that OTM can increase PCC in non-traumatised teeth to a greater extent than that related to physiological ageing.^74^^,^^75^^,^^76^^,^^77^ The differences reported in the literature probably relate to variables including a history of trauma, factors related to OTM (type, duration, force levels and force vectors) and genetic predisposition.^68^ If pulp necrosis occurs in such teeth, endodontic treatment is feasible using contemporary endodontic protocols.^73^^,^^78^^,^^79^ However, if the presenting complaint is only tooth discolouration, then external bleaching strategies can be implemented.^78^^,^^79^^,^^80^

## Orthodontic effects on the vital pulp

The literature review above has established that a history of previous trauma and/or PCC may predispose a tooth to pulpal necrosis after OTM.^13^^,^^14^^,^^15^^,^^16^ Further, because of continuing PCC, subsequent injuries, including OTM,^13^^,^^15^^,^^56^ may affect or sever the vascular supply at the narrowing apical foramen.^48^^,^^55^ Additionally, Andreasen^48^ speculated that the pulp vessels at the apical foramen may be ‘strangulated' due to continued hard tissue formation. This should not be confused with the disproven ‘strangulation theory' of pulp necrosis subsequent to pulpal inflammation;^81^ rather, it could be considered as a compressive obstructive affect. Hence, a brief discussion of the physiological and anatomical effects of OTM on the pulp are important.

### Physiological effects

Orthodontic forces have been reported to cause a wide range of physiological effects on the pulp.^2^ These include initial decreased pulpal blood flow; reactive hyperaemia; a large and coordinated sprouting of pulpal blood vessels and nerve endings; vascular dilation and congestion; increased pulpal cellular responses; pulpal fibrosis and calcifications; odontoblast aspiration and disruption of the odontoblast layer; vacuolisation; vascular degeneration; interference with pulpal sensibility; increase in the expression or activity levels of certain enzymes and neuropeptides associated with inflammation; as well as reversible pulpitis.^2^

**Fig. 3:**
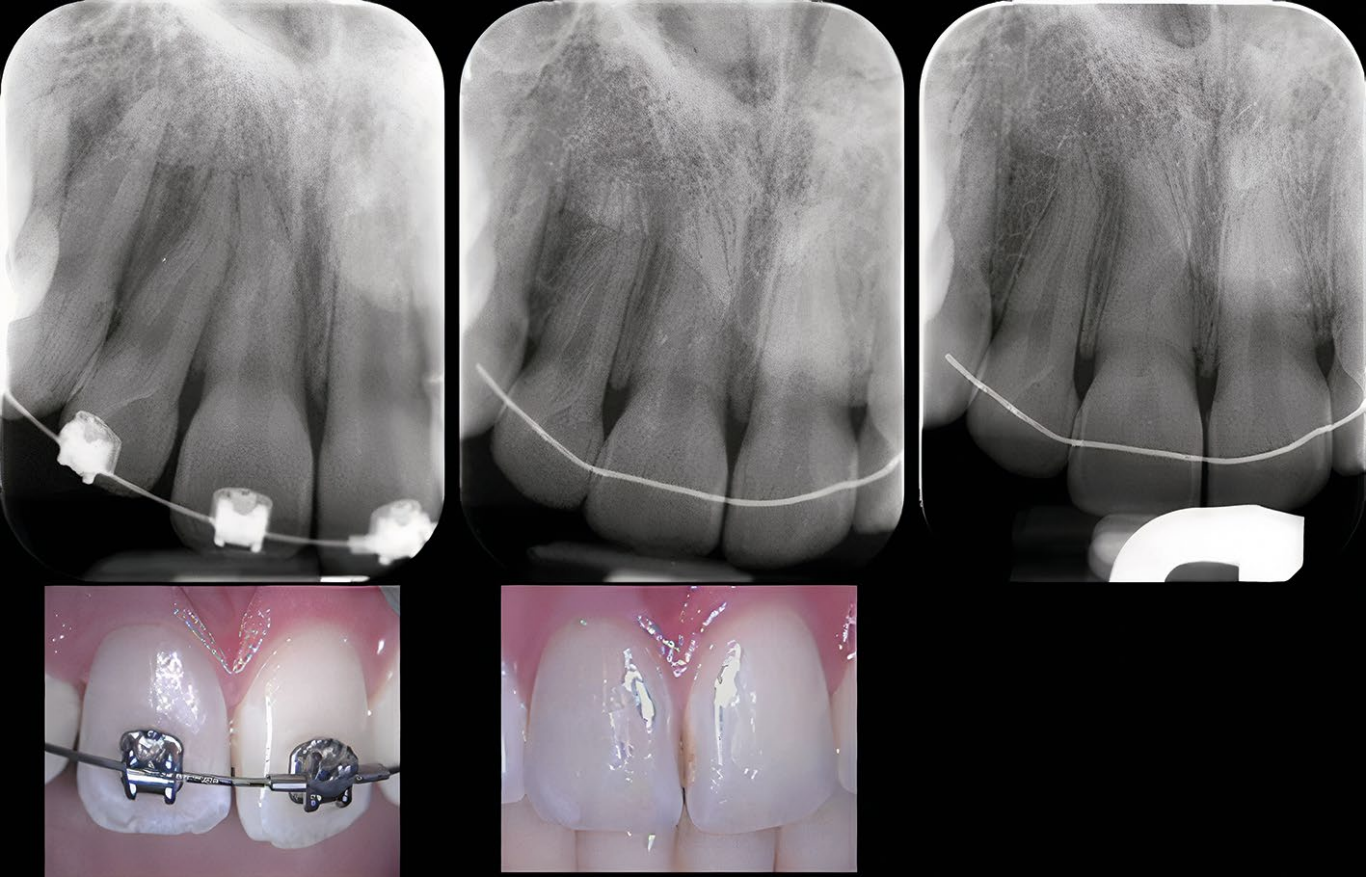
The maxillary right central incisor discoloured during OTM but the pulp was normally responsive to CO_2_ sensibility testing; subsequent reviews showed PCC and improvement in colour. Periapical radiographs at presentation (top left), five-month review (top centre), and 4.3 years (top right). Photographs on presentation (bottom left) and at five-month review (bottom right). Images courtesy of Dr Vijay John

Similarly, a series of recent systematic reviews came to the same conclusions.^7^^,^^8^^,^^17^^,^^74^^,^^75^^,^^76^^,^^82^^,^^83^ Importantly, these responses appeared to be temporary and transient, and usually reversible^74^^,^^75^^,^^76^^,^^82^^,^^83^ if the forces were within the physiological limits for the particular clinical situation.^75^^,^^84^^,^^85^ A systematic review^83^ concluded that OTM did not routinely cause pulpal necrosis; although, other potential co-factors were not evaluated in the review, including a history of trauma and PCC. Consequently, the authors recommended the application of ‘low' orthodontic forces.^83^ A vital pulp traumatised during OTM may manifest clinically as a grey discolouration but with an appropriate rest period may subsequently survive (Fig. 3).

However, despite the above documented adverse pulpal effects of OTM, there is no evidence that the reduction of blood flow is force-dependent.^7^^,^^82^ Nevertheless, it is feasible that the duration, magnitude, vectors and type (especially intrusion)^69^^,^^75^^,^^76^^,^^86^ of OTM would increase the risks to a pulp,^80^^,^^87^ particularly when compromised by other factors such as age,^88^^,^^89^ which reflects the anatomical influence of a decrease in natural apical foramen size and canal dimensions due to progressive calcification.

### Anatomical aspects

**Fig. 4:**
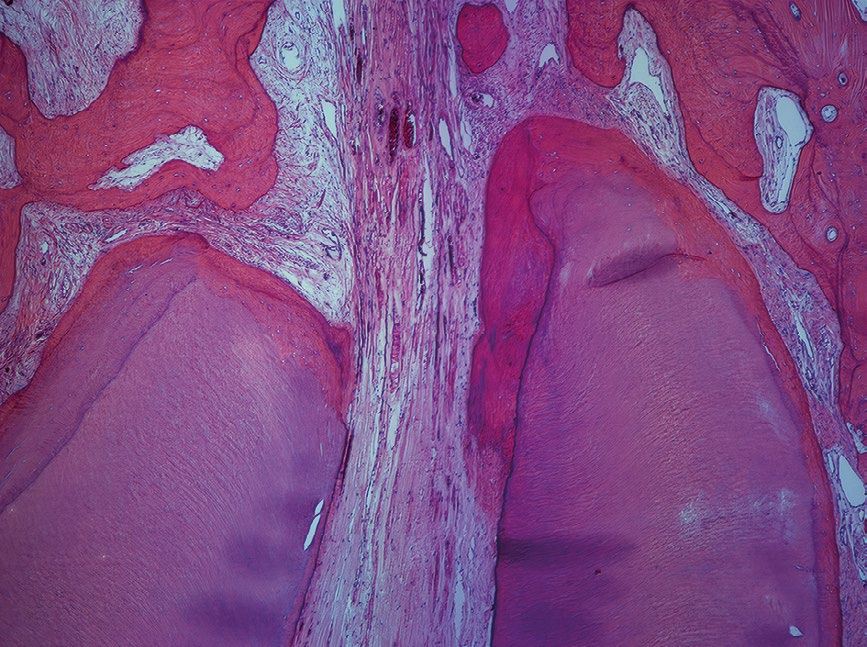
Root apex and surrounding supporting tissues of a human maxillary central incisor (H&E × 40 mag) showing many blood vessels in the NVB, some with red blood cells in their lumens. The fibres in the PDL space seem to be in a very close relationship with the NVB but without affecting its continuity. Image courtesy of Dr Teck Hong Oh^92^

**Fig. 5:**
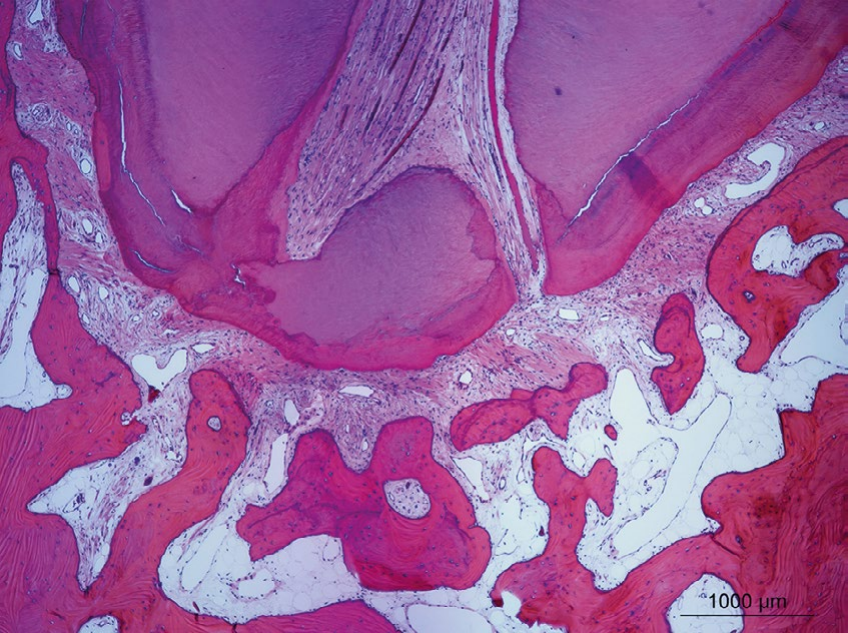
Root apex of mandibular left central incisor with bifurcation of the canal at the apex (H&E × 40 mag). Image courtesy of Dr Teck Hong Oh^92^

**Fig. 6:**
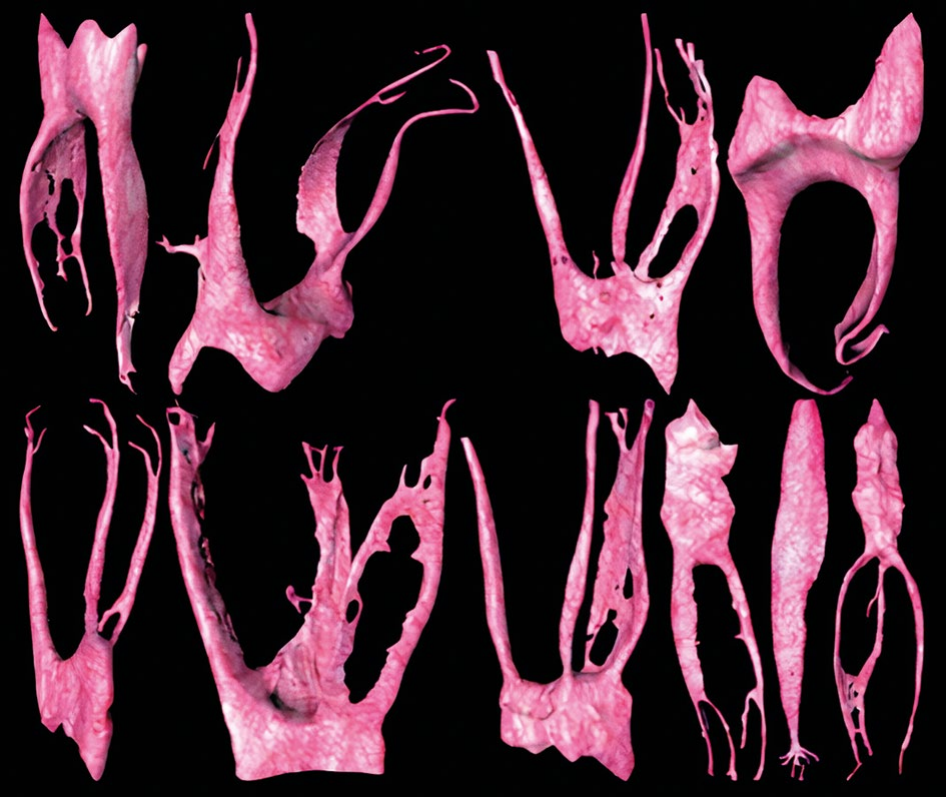
3D reconstructions of different teeth acquired with micro-CT technology depicting the complexity of the apical canal. Image reproduced with permission from Versiani *et al.*, ‘Anatomical complexities affecting root canal preparation: a narrative review', *Australian Dental Journal,* Vol 68, 2023^93^

Almost a century ago, Coolidge^90^ reported that the pulp tissue of a tooth entered the root canal in a large bundle of blood vessels, nerves and connective tissue. This arrangement of the neurovascular bundle (NVB) being continuous between the root canal space and the periapical tissues has been confirmed in subsequent research using contemporary histological techniques (Fig. 4).^91^^,^^92^ While the complexity of the apical root canal anatomy is implied in histological specimens (Fig. 5), three dimensional anatomical studies demonstrate the tortuous and variable nature of the root canal system of teeth, especially at the apical terminus (Fig. 6).^93^

**Fig. 7:**
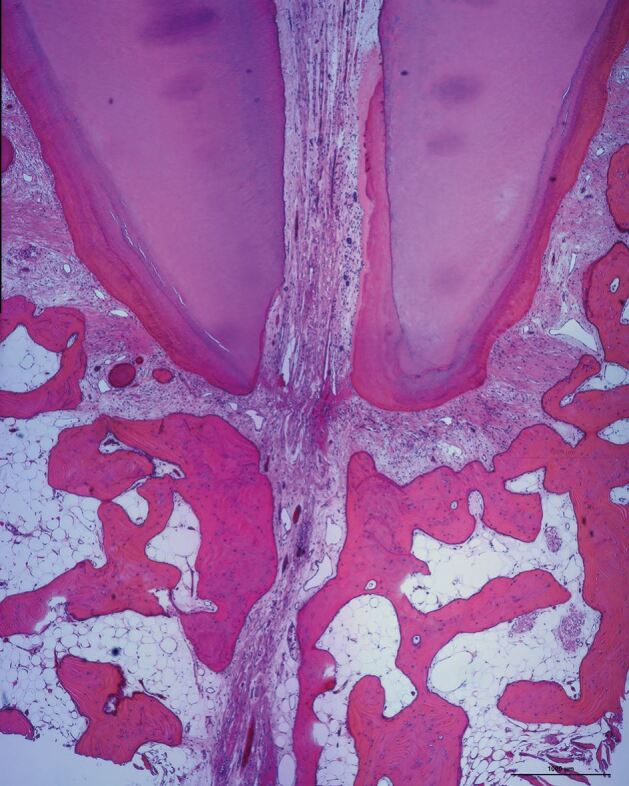
Example of the shift of the apical foramen by resorption of dentine and cementum on the left surface and apposition of cementum on the other surface. Mandibular lateral incisor from a 71-year-old cadaver (H&E x 40 mag). Image courtesy Dr Teck Hong Oh^92^

Such anatomical evidence indicates that the apical foramina vary in size, shape and location, which implies that OTM will result in different force vectors and magnitudes on the NVBs at each foramen of each individual root canal of each individual tooth. Anatomical variations of the apical foramen or the NVB may explain variations in pulpal responses with OTM.^16^^,^^94^ Horizontal forces acting on the root apices of tooth roots during OTM may result in the NVB exerting pressure on one side of the apical foramen leading to root resorption in that region.^95^ Then, on the opposite side of the root apex, cementum may be laid down, with an overall effect of the apical foramen being relocated (Fig. 7).^95^ However, this will depend on the position of the apical foramina, so it is unlikely that all teeth will be affected in exactly the same way. Nevertheless, such images (Fig. 4, Fig. 5, Fig. 6, Fig. 7) allow us to visualise the various apical displacement forces and vectors acting on the apical NVB leading to compression or stretching of the blood vessels and the initial reduction in the blood flow, ultimately compensated for by the reactive hyperaemia.^69^^,^^96^

Another effect of the apical pressure or tension during OTM is the reported increase in response threshold to electric pulp testing.^97^ This may relate to the anatomical relationship between the apical foramen and the NVB, or it may be due to hypoxia of the pulpal Aδ fibres.^97^ However, sensibility testing with cold seems not to be affected,^97^^,^^98^ for which there is currently no explanation.

## Recommendations

The clinically relevant question arising from the interactions between dental trauma and orthodontic treatment relates to the timing of orthodontic treatment for teeth with a history of trauma. This includes clinical scenarios where the trauma has occurred before the commencement of a course of orthodontic care, or where the trauma occurs during the OTM. While it is known that repair of resorptive defects due to OTM occurs rapidly after cessation of forces in teeth with no history of trauma,^99^^,^^100^^,^^101^ intermittent^52^^,^^53^^,^^102^^,^^103^^,^^104^^,^^105^^,^^106^^,^^107^ and light^2^^,^^16^^,^^20^^,^^57^ orthodontic forces may minimise root resorption and pulpal complications. Unfortunately, this available literature is vague and variable in how long the delay period should be before commencing or re-commencing OTM, with a broad range of rest periods suggested for non-trauma influenced OTM.

**Fig. 8:**
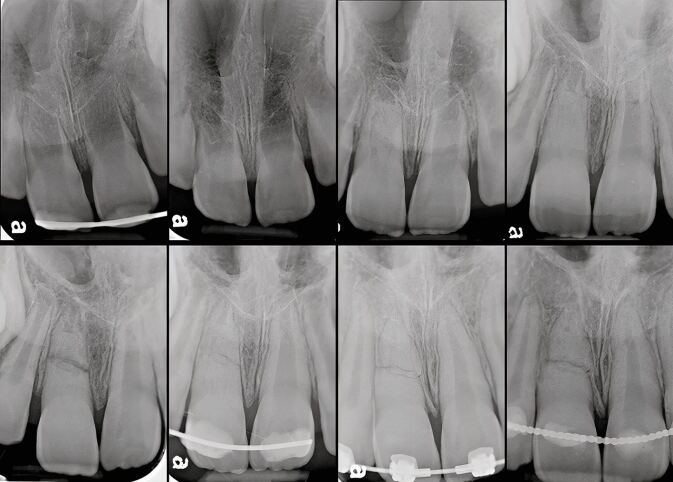
Complex case of a young child suffering avulsion of a right maxillary central incisor that underwent PCC, then transverse root fracture, as well as OTM. Top row, from left: avulsed tooth stored in milk for one hour then replanted and splinted; five weeks later at splint removal; at seven months PCC is advanced; at 18 months PCC and continued root development evident. Bottom row from left: at 22 months, second trauma sustained resulting in transverse root fracture; repositioned and splinted; at around 5.5 years, the patient commenced OTM; at 6 years during OTM, normal appearance; at 7 years, OTM completed with minimal displacement of the fracture fragments. Further review planned. Image courtesy of Dr Ilya Belobrov

**Fig. 9:**
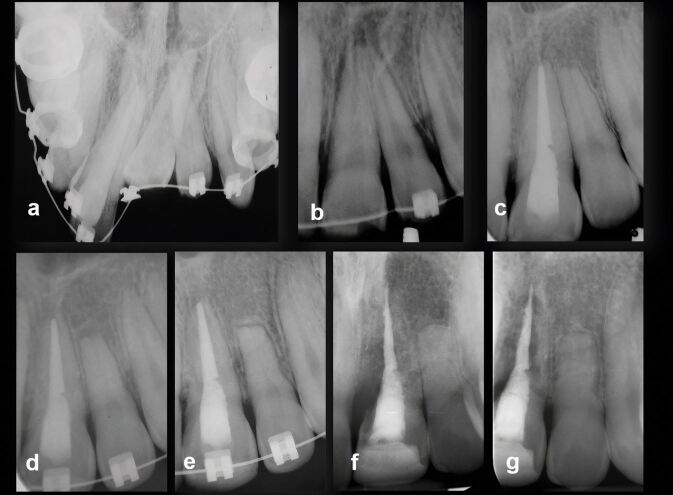
Male patient (13 years old) undergoing a course of OTM suffered a golf club injury resulting in tooth 11 luxated superolabially, 21 intruded with only incisal edge visible and uncomplicated crown fracture, and 22 intruded approximately half the crown height. a) Pre-operative occlusal radiograph. b) March 1997, teeth repositioned and splinted. c) April 1998, 13-month review showing completed root canal treatment of tooth 21 and remodelled tooth 22 undergoing PCC. d) 23-month review, OTM commenced, tooth 22 showing evidence of transient apical breakdown, orthodontist requested to pause OTM. e) 31-month review showing continued PCC of 22 and apical root remodelling. f, g) 22.5-year review showing advanced replacement resorption of 21, and PCC of 22 with normal apical tissues (panels f and g courtesy of Dr Vijay John)

**Fig. 10:**
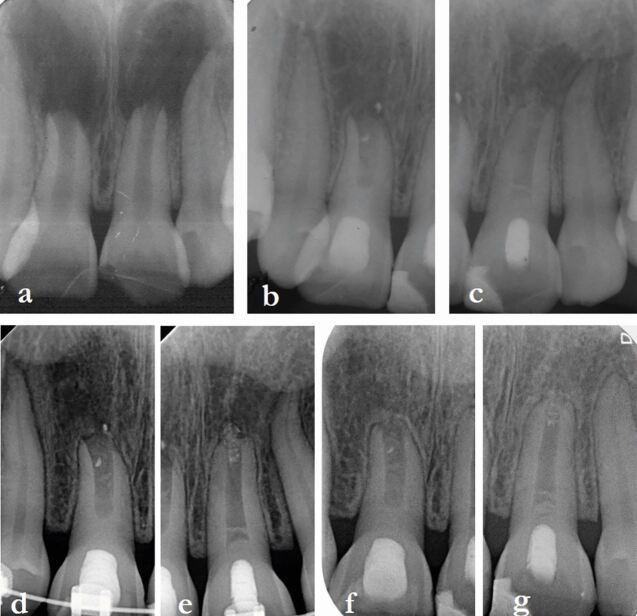
A 17-year-old female patient suffered crown fractures on her maxillary central incisors when she was about nine years old; she could not recall the exact trauma history. She had been asymptomatic since the trauma but had noticed that the teeth were discolouring. The teeth were both diagnosed with necrotic infected pulps and asymptomatic apical periodontitis. The treatment option selected was regenerative endodontic procedures for both teeth. a) Pre-operative periapical radiograph, July 2017. b, c) Tooth 11 and tooth 21, review radiographs February 2020. d, e) Review radiographs, October 2021, ten months after commencement of OTM. f, g) May 2023, review radiographs showing satisfactory biological outcomes for both teeth. Images courtesy of Dr Chee Wei Yeap

Because of the many variables identified in the literature,^2^^,^^23^ it is unlikely that a definitive rest period will ever be recommended. When there is a history of dental trauma, deciding on an appropriate rest period (also known as delay, pause, or waiting periods) is further complicated by the extent and severity of the trauma. However, timing of appropriate rest periods can allow OTM to proceed without further compromise to the traumatised teeth (Fig. 8, Fig. 9, Fig. 10). An additional factor has been the use of clear aligners as an alternative to fixed orthodontic appliances with expanding clinical indications.^108^ Currently, there is no published literature relating outcomes of OTM with clear aligners to a history of dental trauma. However, one study confirmed that initial blood flow changes with clear aligners and fixed appliances eventually return to normal values after OTM with no difference between these two treatment modalities.^109^ While no randomised controlled trials exist, the available evidence indicates that orthodontically induced apical root resorption related to clear aligner treatment is unlikely to be more problematic than with fixed appliance treatment.^110^^,^^111^^,^^112^ Consequently, while it is reasonable to suggest that clear aligners are unlikely to be a risk for teeth with a history of dental trauma, further research in this area is required.

Furthermore, while the degree of trauma sustained is recognised as an important variable,^15^ the degrees of dental trauma injuries are not clearly defined within the literature. Rather than attempting to classify into vague groups such as ‘mild', ‘moderate', or ‘severe', rest period recommendations should recognise a continuum of trauma possibilities with no definitive discriminators.^2^

Therefore, logically, the decision should be based on the biology of the healing responses of the tissues involved (ie pulp and PDL), and also the type of trauma sustained. In cases of multiple types of trauma (Fig. 8, Fig. 9), rest periods should be based on the most severe of the injuries, generally those compromising the PDL. Histologically, pulp healing is reported to occur within some 1-3 months,^113^ which becomes evident radiographically after approximately 3-6 months.^114^^,^^115^ However, periodontal healing involves three phases ie: 1) blood coagulation and inflammation; 2) new tissue formation; and 3) tissue remodelling.^116^^,^^117^ Crucially, while initial healing with type III collagen occurs within two weeks, tissue remodelling to return the periodontal tissues to their pre-wounded state, replacing the randomly-oriented type III collagen with the more organised and crosslinked type I collagen, can take at least one year.^116^^,^^117^

Hence, the recommendations in Table 1 are based not only on previously published guidelines,^9^^,^^10^^,^^11^^,^^23^^,^^118^^,^^119^ but also on the biological influences of pulpal and periodontal tissues discussed above.

**Table 1:** Recommended waiting periods for OTM after dental trauma. Reproduced from Parashos P, ‘Endodontic-orthodontic interactions: a review and treatment recommendations' *Australian Dental Journal*, Vol 68, 2023, Wiley^2^

Injury*	Period**
Crown and crown/root fractures without pulpal exposure	3 months
Crown and crown/root fractures with pulpal exposure (after partial pulpotomy and radiographic signs of establishment of a hard tissue barrier)	6 months
Mild damage to the periodontium (ie minor displacement. Concussion, subluxation - shorter wait for the former and longer for the latter)	3-6 months
Moderate to severe injury to periodontium (ie moderate-severe displacement: luxations - lateral, extrusive, and intrusive, replantation, transplantation)	1 year
Transverse (intra-alveolar) root fractures	1-2 years^†^
Immature traumatised teeth (await radiographic evidence of continued root growth)	2 years
Teeth with radiographic evidence of trauma-induced inflammatory root resorption (await radiographic evidence of root and bone healing)	1 year
Key: * = There will be variations of degree of trauma within the continuum of injury categories, which should be reflected in the observation period chosen by the clinician. Waiting periods are conditional on basic treatment needs being addressed subsequent to the trauma and before the hiatus, for example, traumatically intruded teeth should first be repositioned and stabilised either surgically, orthodontically, or by allowing spontaneous re-eruption depending on the specific case. Interdisciplinary endodontic-orthodontic liaison is highly recommended. If OTM is already in progress, wires may be removed to cease active tooth movement facilitating plaque control, but brackets can remain unless they may compromise PDL and pulpal healing. ** = With more severe injury, a longer waiting/observation period is recommended. Radiographic reviews should be conducted at 6, 12 and 24 months within these periods. Refer to the narrative for further details. ^†^ = Generally, one year is sufficient if the pulp remains vital with no concommitant periodontal injuries (eg extrusive luxation of the coronal fragment).	

## Conclusions

The existing literature base confirms that there are biologically relevant interactions between orthodontics and endodontics, especially when impacted by dental trauma. This review has identified the pulpal and periodontal consequences of trauma and the effects of OTM on these interactions. The broad heterogeneity of the existing literature base is evident, complicating definitive conclusions without continued research to add to the understanding of orthodontic-endodontic interface.

Generally, during OTM, traumatised teeth appear to be more susceptible to pulpal and periapical changes, but the responses of these tissues will depend on the type and the continuum of trauma severity possibilities, as well as the variability of the factors related to OTM. When a dental injury is sustained before or during OTM, the orthodontic rest period depends on the severity of the injury, and best-available evidence-based guidelines should be followed.

## References

[1] Hamilton R S, Gutmann J L. Endodontic-orthodontic relationships: a review of integrated treatment planning challenges. *Int Endod J* 1999; **32:** 343-360. 10.1046/j.1365-2591.1999.00252.x 10551108

[2] Parashos P. Endodontic-orthodontic interactions: a review and treatment recommendations. *Aust Dent J* 2023; **68:** 66-81. 10.1111/adj.12996 37961018

[3] Atack N E. The orthodontic implications of traumatized upper incisor teeth. *Dent Update* 1999; **26:** 432-437. 10.12968/denu.1999.26.10.432 10765786

[4] Stuteville O H. Injuries caused by orthodontic forces and the ultimate results of these injuries. *Am J Orthod Oral Surg* 1938; **24:** 103-119.

[5] Stanley H R, Weisman M I, Michanowicz A E, Bellizzi R. Ischemic infarction of the pulp: Sequential degenerative changes of the pulp after traumatic injury. *J Endod* 1978; **4:** 325-335. 10.1016/S0099-2399(78)80229-5 33222

[6] Rotstein I, Engel G. Conservative management of a combined endodontic-orthodontic lesion. *Endod Dent Traumatol* 1991; **7:** 266-269. 10.1111/j.1600-9657.1991.tb00215.x 1820860

[7] Javed F, Al-Kheraif A A, Romanos E B, Romanos G E. Influence of orthodontic forces on human dental pulp: A systematic review. *Arch Oral Biol* 2015; **60:** 347-356. 10.1016/j.archoralbio.2014.11.011 25463910

[8] Duarte P H M, Weissheimer T, Michel C H T, Só G B, da Rosa R A, Só M V R. Do orthodontic movements of traumatized teeth induce dental pulp necrosis? A systematic review. *Clin Oral Investig* 2023; **27:** 4117-4129. 10.1007/s00784-023-05102-2 37335397

[9] Kindelan S A, Day P F, Kindelan J D, Spencer J R, Duggal M S. Dental trauma: an overview of its influence on the management of orthodontic treatment. Part 1. *J Orthod* 2008; **35:** 68-78. 10.1179/146531207225022482 18525070

[10] Duggal M S, Kindelan J, Nazzal H. Upper incisor trauma and the orthodontic patient - principles of management. *Semin Orthod* 2015; **21:** 59-70.

[11] Bakkari A, Bin Salamah F. Updated guidelines for the orthodontic management of traumatized and endodontically treated teeth: A Review Study. *Cureus* 2022; **14:** e28943. 10.7759/cureus.28943 PMC9547618 36237800

[12] Brin I, Ben-Bassat Y, Heling I, Engelberg A. The influence of orthodontic treatment on previously traumatized permanent incisors. *Eur J Orthod* 1991; **13:** 372-377. 10.1093/ejo/13.5.372 1748184

[13] Bauss O, Röhling J, Rahman A, Kiliaridis S. The effect of pulp obliteration on pulpal vitality of orthodontically intruded traumatized teeth. *J Endod* 2008; **34:** 417-420. 10.1016/j.joen.2008.01.006 18358887

[14] Bauss O, Röhling J, Sadat-Khonsari R, Kiliaridis S. Influence of orthodontic intrusion on pulpal vitality of previously traumatized maxillary permanent incisors. *Am J Orthod Dentofacial Orthop* 2008; **134:** 12-17. 10.1016/j.ajodo.2006.07.033 18617098

[15] Bauss O, Röhling J, Meyer K, Kiliaridis S. Pulp vitality in teeth suffering trauma during orthodontic therapy. *Angle Orthod* 2009; **79:** 166-171. 10.2319/010708-7.1 19123692

[16] Bauss O, Schäfer W, Sadat-Khonsari R, Knösel M. Influence of orthodontic extrusion on pulpal vitality of traumatized maxillary incisors. *J Endod* 2010; **36:** 203-207. 10.1016/j.joen.2009.10.025 20113775

[17] Von Böhl M, Ren Y, Fudalej P S, Kuijpers-Jagtman A M. Pulpal reactions to orthodontic force application in humans: a systematic review. *J Endod* 2012; **38:** 1463-1469. 10.1016/j.joen.2012.07.001 23063219

[18] Brezniak N, Wasserstein A. Root resorption after orthodontic treatment: Part 2. Literature review. *Am J Orthod Dentofac Orthop* 1993; **103:** 138-146. 10.1016/S0889-5406(05)81763-9 8427218

[19] Lopatiene K, Dumbravaite A. Risk factors of root resorption after orthodontic treatment. *Stomatologija* 2008; **10:** 89-95. 19001842

[20] Weltman B, Vig K W L, Fields H W, Shanker S, Kaizar E E. Root resorption associated with orthodontic tooth movement: A systematic review. *Am J Orthod Dentofacial Orthop* 2010; **137:** 462-476. 10.1016/j.ajodo.2009.06.021 20362905

[21] Beck V J, Stacknik S, Chandler N P, Farella M. Orthodontic tooth movement of traumatised or root-canal-treated teeth: a clinical review. *N Z Dent J* 2013; **109:** 6-11. 23923150

[22] Fields H W, Christensen J R. Orthodontic procedures after trauma. *J Endod* 2013; **39:** 78-87. 10.1016/j.joen.2012.10.030 23439050

[23] Morris H T, Campbell R E, Kissling A D, Cully J L, Thikkurissy S. Observation periods before tooth movement in orthodontic patients who have experienced mild-to-moderate dental trauma: a scoping review of current evidence. *J World Fed Orthod* 2022; **11:** 59-68. 10.1016/j.ejwf.2021.12.003 35184986

[24] Phillips J R. Apical root resorption under orthodontic therapy. *Angle Orthod* 1955; **25:** 1-22.

[25] Goldson L, Henrikson C O. Root resorption during Begg treatment; a longitudinal roentgenologic study. *Am J Orthod* 1975; **68:** 55-66. 10.1016/0002-9416(75)90159-1 1056144

[26] Hines F B. A radiographic evaluation of the response of previously avulsed teeth and partially avulsed teeth to orthodontic movement. *Am J Orthod* 1979; **75:** 1-19. 10.1016/0002-9416(79)90135-0 283691

[27] Malmgren O, Goldson L, Hill C, Orwin A, Petrini L, Lundberg M. Root resorption after orthodontic treatment of traumatized teeth. *Am J Orthod* 1982; **82:** 487-491. 10.1016/0002-9416(82)90317-7 6961819

[28] Linge B O, Linge L. Apical root resorption in upper anterior teeth. *Eur J Orthod* 1983; **5:** 173-183. 10.1093/ejo/5.3.173 6578039

[29] Linge L, Linge B O. Patient characteristics and treatment variables associated with apical root resorption during orthodontic treatment. *Am J Orthod Dentofac Orthop* 1991; **99:** 35-43. 10.1016/S0889-5406(05)81678-6 1986524

[30] Levander E, Malmgren O, Eliasson S. Evaluation of root resorption in relation to two orthodontic treatment regimes. A clinical experimental study. *Eur J Orthod* 1994; **16:** 223-228. 10.1093/ejo/16.3.223 8062862

[31] Mandall N A, Lowe C, Worthington H V *et al*. Which orthodontic archwire sequence? A randomized clinical trial. *Eur J Orthod* 2006; **28:** 561-566. 10.1093/ejo/cjl030 17041083

[32] Makedonas D, Lund H, Gröndahl K, Hansen K. Root resorption diagnosed with cone beam computed tomography after 6 months of orthodontic treatment with fixed appliance and the relation to risk factors. *Angle Orthod* 2012; **82:** 196-201. 10.2319/112810-691.1 PMC8867938 21827236

[33] Michielsens H, Decreus J, Begnoni G *et al*. Performance of the Malmgren index for assessing root resorption on 2D vs 3D radiographs: a pilot study. *Healthcare (Basel)* 2023; **11:** 1860. 10.3390/healthcare11131860 PMC10340771 37444694

[34] El-Angbawi, Yassir Y A, McIntyre G T, Revie G F, Bearn D R. A randomized clinical trial of the effectiveness of 0.018-inch and 0.022-inch slot orthodontic bracket systems: part 3—biological side-effects of treatment. *Eur J Orthod* 2019; **41:** 154-164. 10.1093/ejo/cjy039 30007330

[35] Smeyers F, Fivez S, Van Gorp G *et al*. Evolution of root length throughout orthodontic treatment in maxillary incisors with previous history of dental trauma: a longitudinal controlled trial. *Clin Oral Investig* 2022; **26:** 7179-7190. 10.1007/s00784-022-04679-4 35982348

[36] Acevedo-Mascarúa A E, Torres-Rosas R, Pérez-Cervera Y *et al*. External apical root resorption in orthodontic patients who practice combat sports: A case-control observational pilot study. *Medicina (Kaunas)* 2022; **58:** 1342. 10.3390/medicina58101342 PMC9610488 36295503

[37] Keinan D, Asbi T, Shalish M, Slutzky-Goldberg I. An assessment of the effects of orthodontic treatment after apexification of traumatized immature permanent teeth: a retrospective study. *J Endod* 2022; **48:** 96-101. 10.1016/j.joen.2021.09.012 34619170

[38] Chaniotis A. Orthodontic movement after regenerative endodontic procedure: case report and long-term observations. *J Endod* 2018; **44:** 432-437. 10.1016/j.joen.2017.11.008 29306536

[39] Jawad Z, Bates C, Duggal M, Nazzal H. Orthodontic management of a non-vital immature tooth treated with regenerative endodontics: a case report. *J Endod* 2018; **45:** 289-295. 10.1080/14653125.2018.1501935 30022713

[40] Alharbi M A, Lee S-M. Long-term follow-up for immature teeth treated with regenerative endodontic procedures that underwent orthodontic treatment. *Eur Endod J* 2021; **6:** 242-246. 10.14744/eej.2020.29591 PMC8461485 34650020

[41] Chaniotis A, Chanioti A. Long-term complications of previously successful regenerative endodontic procedures after orthodontic movement: a report of 3 different complications after 4: 8, and 11 years. *J Endod* 2022; **48:** 951-960. 10.1016/j.joen.2022.04.002 35405157

[42] Dawood H M, Kroeger A, Chavda V, Chapple I L C, Kebschull M. Under pressure - mechanisms and risk factors for orthodontically induced inflammatory root resorption: a systematic review. *Eur J Orthod* 2023; **45:** 612-626. 10.1093/ejo/cjad011 PMC10505745 37366151

[43] Alam M K, Awawdeh M, Aljhani A S *et al*. Impact of dental trauma on orthodontic parameters - a systematic review and meta-analysis. *Children (Basel)* 2023; **10:** 885. 10.3390/children10050885 PMC10217624 37238433

[44] Ottofy L. Observations on the causes of erosion. *Dent Cosmos* 1905; **47:** 71-78.

[45] Smyth K C. Obliteration of the pulp of a permanent incisor at the age of 13-9/12 years. *Dent Rec* 1950; **70:** 218-219. 24539302

[46] Archer W H. Replantation of an accidentally extracted erupted partially formed mandibular second premolar. *Oral Surg Oral Med Oral Pathol* 1952; **5:** 256-258. 10.1016/0030-4220(52)90123-0 14911168

[47] Andreasen J O, Hjørting-Hansen E. Replantation of teeth I: Radiographic and clinical study of 110 human teeth replanted after accidental loss. *Acta Odont Scand* 1966; **24:** 263-286. 10.3109/00016356609028222 5225449

[48] Andreasen J O. Luxation of permanent teeth due to trauma. *Scand J Dent Res* 1970; **78:** 273-286. 10.1111/j.1600-0722.1970.tb02074.x 5273695

[49] Jacobsen I, Kerekes K. Long-term prognosis of traumatized permanent anterior teeth showing calcifying processes in the pulp cavity. *Scand J Dent Res* 1977; **85:** 588-598. 10.1111/j.1600-0722.1977.tb02119.x 272723

[50] Andreasen F M, Yu Z, Thomsen B L, Andersen P K. Occurrence of pulp canal obliteration after luxation injuries in the permanent dentition. *Endod Dent Traumatol* 1987; **3:** 103-115. 10.1111/j.1600-9657.1987.tb00611.x 3476298

[51] Andreasen F M. Pulpal healing after luxation injuries and root fracture in the permanent dentition. *Endod Dent Traumatol* 1989; **5:** 111-131. 10.1111/j.1600-9657.1989.tb00348.x 2699588

[52] Acar A, Canyürek Ü, Kocaaga M, Erverdi N. Continuous vs. discontinuous force application and root resorption. *Angle Orthod* 1999; **69:** 159-164. 10.1043/0003-3219(1999)069<0159:CVDFAA>2.3.CO;2 10227557

[53] Sawicka M, Bedini R, Wierzbicki P M, Pameijer C H. Interrupted orthodontic force results in less root resorption than continuous force in human premolars as measured by microcomputed tomography. *Folia Histochem Cytobiol* 2014; **52:** 289-296. 10.5603/FHC.a2014.0037 25530465

[54] Robertson A, Andreasen F M, Bergenholtz G, Andreasen J O, Norén J G. Incidence of pulp necrosis subsequent to pulp canal obliteration from trauma of permanent incisors. *J Endod* 1996; **22:** 557-560. 10.1016/S0099-2399(96)80018-5 9198446

[55] Robertson A. A retrospective evaluation of patients with uncomplicated crown fractures and luxation injuries. *Endod Dent Traumatol* 1998; **14:** 245-256. 10.1111/j.1600-9657.1998.tb00848.x 9972156

[56] Jacobsen I, Zachrisson B U. Repair characteristics of root fractures in permanent anterior teeth. *Scand J Dent Res* 1975; **83:** 355-364. 10.1111/j.1600-0722.1975.tb00449.x 1060162

[57] Popp T W, Artun J, Linge L. Pulpal response to orthodontic tooth movement in adolescents: a radiographic study. *Am J Orthod Dentofacial Orthop* 1992; **101:** 228-233. 10.1016/0889-5406(92)70091-N 1539549

[58] Heithersay G S. Life cycles of traumatized teeth: long-term observations from a cohort of dental trauma victims. *Aust Dent J* 2016; **61:** 120-127. 10.1111/adj.12403 26923453

[59] Delivanis H P, Sauer G J. Incidence of canal calcification in the orthodontic patient. *Am J Orthod* 1982; **82:** 58-61. 10.1016/0002-9416(82)90547-4 6961778

[60] Cwyk F, Saint-Pierre F, Tronstad L. Endodontic implications of orthodontic tooth movement. *J Dent Res* 1984; **63:** 286.

[61] Woloshyn H, Artun J, Kennedy D B, Joondeph D R. Pulpal and periodontal reactions to orthodontic alignment of palatally impacted canines. *Angle Orthod* 1994; **64:** 257-264. 10.1043/0003-3219(1994)064<0257:PAPRTO>2.0.CO;2 7978520

[62] Ertas E T, Veli I, Akin M, Ertas H, Atici M Y. Dental pulp stone formation during orthodontic treatment: A retrospective clinical follow-up study. *Niger J Clin Pract* 2017; **20:** 37-42. 10.4103/1119-3077.164357 27958244

[63] Jena D, Balakrishna K, Singh S, Naqvi Z A, Lanje A, Arora N. A retrospective analysis of pulp stones in patients following orthodontic treatment. *J Contemp Dent Pract* 2018; **19:** 1095-1099. 30287710

[64] Korkmaz Y N, Aydin Z U, Sarioglu B. Orthodontic treatment and pulp stone formation: is there a relationship? *Clin Exp Health Sci* 2019; **9:** 340-344.

[65] Venkatesh S, Ajmera S, Ganeshkar S V. Volumetric pulp changes after orthodontic treatment determined by cone-beam computed tomography. *J Endod* 2014; **40:** 1758-1763. 10.1016/j.joen.2014.07.029 25224263

[66] Afsari E, Niksolat E, Ostovar F, Karimi S. Comparison of abundance of premolar and molar pulp stones before and after orthodontic treatment using panoramic radiography. *Front Dent* 2021; **18:** 22 10.18502/fid.v18i22.6932 PMC9355851 35965700

[67] Babanouri N, Sahmeddini S, Khoshmakani M R. Effects of orthodontic treatment on pulp stone formation: a retrospective study. *Biomed Res Int* 2023; DOI: 10.1155/2023/7381610. 10.1155/2023/7381610 PMC10121340 37090191

[68] Ramirez I, Kirschneck C, Corrêa Silva-Sousa A *et al*. The investigation of WNT6 and WNT10A single nucleotide polymorphisms as potential biomarkers for dental pulp calcification in orthodontic patients. *PLos ONE* 2023; **18:** e0288782. 10.1371/journal.pone.0288782 PMC10420345 37566620

[69] Stenvik A, Mjör I A. Pulp and dentine reactions to experimental tooth intrusion. *Am J Orthod* 1970; **57:** 370-385. 10.1016/s0002-9416(70)90219-8 5265007

[70] Han G, Hu M, Zhang Y, Jiang H. Pulp vitality and histologic changes in human dental pulp after the application of moderate and severe intrusive orthodontic forces. *Am J Orthod Dentofacial Orthop* 2013; **144:** 518-522. 10.1016/j.ajodo.2013.05.005 24075659

[71] Lazzaretti D N, Bortoluzzi G S, Fernandes L F, Rodriguez R, Grehs R A, Hartmann M S. Histologic evaluation of human pulp tissue after orthodontic intrusion. *J Endod* 2014; **40:** 1537-1540. 10.1016/j.joen.2013.10.039 25115659

[72] Ramazanzadeh B A, Sahhafian A A, Mohtasham N, Hassanzadeh N, Jahanbin A, Shakeri M T. Histological changes in human dental pulp following application of intrusive and extrusive orthodontic forces. *J Oral Sci* 2009; **51:** 109-115. 10.2334/josnusd.51.109 19325207

[73] Goga R, Chandler N P, Oginni A O. Pulp stones: a review. *Int Endod J* 2008; **41:** 457-468. 10.1111/j.1365-2591.2008.01374.x 18422587

[74] de Andrade Vieira W, Oliveira M B, de Souza Machado L, Cericato G O, Lima I F P, Paranhos L R. Pulp changes from rapid maxillary expansion: A systematic review. *Orthod Craniofac Res* 2021; **25:** 320-335. 10.1111/ocr.12556 34874608

[75] Vitali F C, Cardoso I V, Mello F W *et al*. Association between orthodontic force and dental pulp changes: a systematic review of clinical and radiographic outcomes. *J Endod* 2022; **48:** 298-311. 10.1016/j.joen.2021.11.018 34890594

[76] Huokuna J, Loimaranta V, Laine M A, Svedström-Oristo A-L. Adverse effects of orthodontic forces on dental pulp. Appearance and character. A systematic review. *Acta Odontol Scand* 2023; **81:** 267-277. 10.1080/00016357.2022.2137232 36436210

[77] Farias Z, Sousa J, Faria C, Vieira J, Sobral A, Silveira M. Pulpal calcifications in orthodontically moved teeth: Scoping review. *J Clin Exp Dent* 2023; **15:** 773-780. 10.4317/jced.60777 PMC10550074 37799748

[78] McCabe P S, Dummer P M H. Pulp canal obliteration: an endodontic diagnosis and treatment challenge. *Int Endod J* 2012; **45:** 177-197. 10.1111/j.1365-2591.2011.01963.x 21999441

[79] Van der Vyver P J, Vorster M, Jonker C H, Potgieter N. Calcific metamorphosis - a review of literature and clinical management. *S Afr Dent J* 2020; **75:** 316-322.

[80] Vinagre A, Castanheira C, Messias A, Palma P J, Ramos J C. Management of pulp canal obliteration - systematic review of case reports. *Medicine (Kaunas)* 2021; **57:** 1237. 10.3390/medicina57111237 PMC8625069 34833455

[81] Van Hassell H J. Physiology of the human dental pulp. *Oral Surg Oral Med Oral Pathol* 1971; **32:** 126-134. 10.1016/0030-4220(71)90258-1 5281545

[82] Scherer I, Tzanetakis G N, Eliades T, Koletsi D. Changes in the pulp tissue complex induced by orthodontic forces: Is there a need for concern? A systematic review and meta-analysis of RCTs and prospective clinical trials. *Oral Health Prev Dent* 2022; **20:** 433-448. 10.3290/j.ohpd.b3556039 PMC11640781 36346338

[83] Weissheimer T, Silva E J N L, Pinto K P, Só G B, Rosa R A, Só M V R. Do orthodontic tooth movements induce pulp necrosis? A systematic review. *Int Endod J* 2021; **54:** 1246-1262. 10.1111/iej.13523 33780015

[84] Krishnan V, Davidovitch Z. On a path to unfolding the biological mechanisms of orthodontic tooth movement. *J Dent Res* 2009; **88:** 597-608. 10.1177/0022034509338914 19641146

[85] Henneman S, Von den Hoff J W, Maltha J C. Mechanobiology of tooth movement. *Eur J Orthod* 2008; **30:** 299-306. 10.1093/ejo/cjn020 18540017

[86] Hamersky P A, Weimer A D, Taintor J F. The effect of orthodontic force application on the pulpal tissue respiration rate in the human premolar. *Am J Orthod* 1980; **77:** 368-378. 10.1016/0002-9416(80)90103-7 6928739

[87] Ersahan S, Sabuncuoğlu F A. Effects of magnitude of intrusive force on pulpal blood flow in maxillary molars. *Am J Orthod Dentofacial Orthop* 2015; **148:** 83-89. 10.1016/j.ajodo.2015.02.026 26124031

[88] Daud S, Nambiar P, Hossain M Z, Rahman M R A, Bakri M M. Changes in cell density and morphology of selected cells of the ageing human dental pulp. *Gerodontology* 2016; **33:** 315-321. 10.1111/ger.12154 25266855

[89] Ersahan S, Sabuncuoğlu F A. Effect of age on pulpal blood flow in human teeth during orthodontic movement. *J Oral Sci* 2018; **60:** 446-452. 10.2334/josnusd.17-0316 30249934

[90] Coolidge E D. Anatomy of the root apex in relation to treatment problems. *J Am Dent Assoc* 1929; **16:** 1456-1465.

[91] Nair P N R. Apical periodontitis: a dynamic encounter between root canal infection and host response. *Periodontol 2000* 1997; **13:** 121-148. 10.1111/j.1600-0757.1997.tb00098.x 9567926

[92] Oh T H. *Anatomy of the Human Tooth Apex and Surrounding Tissues: A Histological Cadaver Study.* Melbourne: The University of Melbourne, 2023. DCD Thesis.

[93] Versiani M A, Martins J N R, Ordinola-Zapata R. Anatomical complexities affecting root canal preparation: a narrative review. *Aust Dent J* 2023; **68:** 5-23. 10.1111/adj.12992 37984802

[94] Guevara M J, McClugage S G Jr. Effects of intrusive forces upon the microvasculature of the dental pulp. *Angle Orthod* 1980; **50:** 129-134. 10.1043/0003-3219(1980)050<0129:EOIFUT>2.0.CO;2 6929168

[95] Kallianpur S. Pulp. *In* Kumar G S (ed) *Orban's Oral Histology and Embryology.* 14th ed. pp 94-94. India: Elsevier, 2015.

[96] Sano Y, Ikawa M, Sugawara J, Horiuchi H, Mitani H. The effect of continuous intrusive force on human pulpal blood flow. *Eur J Orthod* 2002; **24:** 159-166. 10.1093/ejo/24.2.159 12001552

[97] Alomari F A, Al-Habahbeh R, Alsakarna B K. Responses of pulp sensibility tests during orthodontic treatment and retention. *Int Endod J* 2011; **44:** 635-643. 10.1111/j.1365-2591.2011.01865.x 21366625

[98] Hall C J, Freer T J. The effects of early orthodontic force application on pulp test responses. *Aust Dent J* 1998; **43:** 359-361. 10.1111/j.1834-7819.1998.tb00189.x 9848990

[99] Owman-Moll P, Kurol J, Lundgren D. Repair of orthodontically induced root resorption in adolescents. *Angle Orthod* 1995; **65:** 403-410. 10.1043/0003-3219(1995)065<0403:ROOIRR>2.0.CO;2 8702065

[100] Owman-Moll P, Kurol J. The early reparative process of orthodontically induced root resorption in adolescents - location and type of tissue. *Eur J Orthod* 1998; **20:** 727-732. 10.1093/ejo/20.6.727 9926640

[101] Cheng L L, Türk T, Elekdağ-Türk S, Jones A S, Yu Y, Darendeliler M A. Repair of root resorption 4 and 8 weeks after application of continuous light and heavy forces on premolars for 4 weeks: a histology study. *Am J Orthod Dentofacial Orthop* 2010; **138:** 727-734. 10.1016/j.ajodo.2009.01.029 21130331

[102] Harris E F. Root resorption during orthodontic therapy. *Semin Orthod* 2000; **6:** 183-194.

[103] Ballard D J, Jones A S, Petocz P, Darendeliler M A. Physical properties of root cementum: Part 11 Continuous vs intermittent controlled orthodontic forces on root resorption. A microcomputed-tomography study. *Am J Orthod Dentofacial Orthop* 2009; **136:** 8. 10.1016/j.ajodo.2007.07.026 19577132

[104] Aras B, Cheng L L, Turk T, Elekdag-Turk S, Jones A S, Darendeliler M A. Physical properties of root cementum: Part 23. Effects of 2 or 3 weekly reactivated continuous or intermittent orthodontic forces on root resorption and tooth movement: A microcomputed tomography study. *Am J Orthod Dentofacial Orthop* 2012; **141:** 29-37. 10.1016/j.ajodo.2011.07.018 22284296

[105] Mehta S A, Deshmukh S V, Sable R B, Patil A S. Comparison of 4 and 6 weeks of rest period for repair of root resorption. *Prog Orthod* 2017; **18:** 18. 10.1186/s40510-017-0173-1 PMC5511810 28670661

[106] Ozkalaycia N, Karadeniz E I, Elekdag-Turk S, Turk T, Cheng L L, Darendeliler M A. Effect of continuous versus intermittent orthodontic forces on root resorption: A microcomputed tomography study. *Angle Orthod* 2018; **88:** 733-739. 10.2319/012518-68.1 PMC8174074 30124325

[107] Ghaleb S, Tamish N, ElKenany W, Guindi M. The effect of two different types of forces on possible root resorption in relation to dentin phosphoprotein levels: a single-blind, split-mouth, randomized controlled trial. *Prog Orthod* 2021; **22:** 44. 10.1186/s40510-021-00388-y PMC8685187 34927213

[108] Kang F, Wu Y, Cui Y, Yuan J, Hu Z, Zhu X. The displacement of teeth and stress distribution on periodontal ligament under different upper incisors proclination with clear aligner in cases of extraction: a finite element study. *Prog Orthod* 2023; **24:** 38. 10.1186/s40510-023-00491-2 PMC10657915 37981597

[109] Abu Alhaija E S J, Al-Abdallah S Y, Taha N A. A comparative study of initial changes in pulpal blood flow between clear aligners and fixed orthodontic appliances. *Am J Orthod Dentofacial Orthop* 2019; **156:** 603-610. 10.1016/j.ajodo.2018.11.013 31677668

[110] Fang X, Qi R, Liu C. Root resorption in orthodontic treatment with clear aligners: A systematic review and meta-analysis. *Orthod Craniofac Res* 2019; **22:** 259-269. 10.1111/ocr.12337 31323701

[111] Yassir Y A, Nabbat S A, McIntyre G T, Bearn D R. Clinical effectiveness of clear aligner treatment compared to fixed appliance treatment: an overview of systematic reviews. *Clin Oral Investig* 2022; **26:** 2353-2370. 10.1007/s00784-021-04361-1 34993617

[112] Almagrami I, Almashraqi A A, Almaqrami B S *et al*. A quantitative three-dimensional comparative study of alveolar bone changes and apical root resorption between clear aligners and fixed orthodontic appliances. *Prog Orthod* 2023; **24:** 6. 10.1186/s40510-023-00458-3 PMC9968667 36843193

[113] Stanley H R. Pulp capping: conserving the dental pulp - can it be done? Is it worth it? *Oral Surg Oral Med Oral Pathol* 1989; **68:** 628-639. 10.1016/0030-4220(89)90252-1 2682429

[114] Cvek M. A clinical report on partial pulpotomy and capping with calcium hydroxide in permanent incisors with complicated crown fracture. *J Endod* 1978; **4:** 232-237. 10.1016/S0099-2399(78)80153-8 283188

[115] Cvek M, Andreasen J O, Borum M K. Healing of 208 intra-alveolar root fractures in patients aged 7-17 years. *Dent Traumatol* 2001; **17:** 53-62. 10.1034/j.1600-9657.2001.017002053.x 11475947

[116] Gurtner G C, Werner S, Barrandon Y, Longaker M T. Wound repair and regeneration. *Nature* 2008; **453:** 314-321. 10.1038/nature07039 18480812

[117] Smith P C, Martínez C, Martínez J, McCulloch C A. Role of fibroblast populations in periodontal wound healing and tissue remodeling. *Front Physiol* 2019; **10:** 270. 10.3389/fphys.2019.00270 PMC6491628 31068825

[118] Malmgren O, Malmgren B. Orthodontic management of the traumatized dentition. *In* Andreasen J O, Andreasen F M, Andersson L (eds) *Textbook and Color Atlas of Traumatic Injuries to the Teeth*. 5th ed. pp 752-791. New Jersey: Wiley-Blackwell, 2019.

[119] Sandler S, Al-Musfir T, Barry S *et al*. Guidelines for the orthodontic management of the traumatised tooth. *J Orthod* 2021; **48:** 74-81. 10.1177/1465312520977498 PMC8042518 33325314

